# Salt intake and gastric cancer risk according to *Helicobacter pylori* infection, smoking, tumour site and histological type

**DOI:** 10.1038/sj.bjc.6605993

**Published:** 2010-11-16

**Authors:** B Peleteiro, C Lopes, C Figueiredo, N Lunet

**Affiliations:** 1 Department of Hygiene and Epidemiology, Faculty of Medicine, University of Porto, Porto, Portugal; 2 Institute of Public Health, University of Porto (ISPUP), Porto, Portugal; 3 Institute of Molecular Pathology and Immunology, University of Porto (IPATIMUP), Porto, Portugal; 4 Department of Pathology, Faculty of Medicine, University of Porto, Porto, Portugal

**Keywords:** stomach neoplasms, salt intake, smoking, *Helicobacter pylori*, tumour site, histological type

## Abstract

**Background:**

Although salt intake is considered a probable risk factor for gastric cancer, relevant studies have provided heterogeneous results, and the magnitude of the association has not been accurately quantified.

**Methods:**

To quantify gastric cancer risk in relation to dietary salt exposure according to *Helicobacter pylori* infection status and virulence, smoking, tumour site, and histological type, we evaluated 422 gastric cancer cases and 649 community controls. Salt exposure was estimated in the year before the onset of symptoms through: sodium intake (estimated by a food frequency questionnaire (FFQ)); main food items/groups contributing to dietary sodium intake; visual analogical scale for salt intake preference; use of table salt; and duration of refrigerator ownership.

**Results:**

Comparing subjects with the highest with those with the lowest salt exposure (3rd *vs* 1st third), sodium intake (OR=2.01, 95% CI: 1.16–3.46), consumption of food items with high contribution to sodium intake (OR=2.54, 95% CI: 1.56–4.14) and salt intake evaluated by visual analogical scale (OR=1.83, 95% CI: 1.28–2.63) were associated with an increased gastric cancer risk. Subjects owning a refrigerator for >50 years had a lower risk for gastric cancer (OR=0.28, 95% CI: 0.14–0.57). These associations were observed regardless of *H. pylori* infection status and virulence, smoking, tumour site or histological type.

**Conclusion:**

Our results support the view that salt intake is an important dietary risk factor for gastric cancer, and confirms the evidence of no differences in risk according to *H. pylori* infection and virulence, smoking, tumour site and histological type.

The decline in gastric cancer mortality (Coleman *et al*, 1993) has primarily been attributed to a more frequent consumption of fresh products, together with a decrease of salt in food preservation (Howson *et al*, 1986). In 2007, salt and salted/salty foods were classified as probable risk factors for gastric cancer (World Cancer Research Fund/American Institute for Cancer Research, 2007). However, the available evidence is mixed, largely because of the complexity of evaluating salt intake (Dias-Neto *et al*, 2010), but it may also reflect aetiological differences between gastric cancer subtypes, or potential effect modification not accounted for in the analyses. Few studies presented subgroup analyses according to histological type, although a greater influence of environmental factors on the intestinal than the diffuse type has been suggested (Lunet *et al*, 2007; Ladeiras-Lopes *et al*, 2008). A differential association between salt intake and gastric cancers with different topographies may occur, as observed for *Helicobacter pylori* infection (Huang *et al*, 1998; Helicobacter and Cancer Collaborative Group, 2001). Also, a potential synergistic effect of salt and *H. pylori* has been described (Shikata *et al*, 2006), as high dietary salt intake may enhance the deleterious effects of infection (Fox *et al*, 1999; Loh *et al*, 2007). Smokers may have a higher average salt consumption (Chen *et al*, 2002; van den Brandt *et al*, 2003; Shikata *et al*, 2006), possibly potentiating both the above detrimental effects (Tatematsu *et al*, 1975; Charnley and Tannenbaum, 1985).

Therefore, we aimed to quantify the association between dietary salt exposure, assessed by different methods, and gastric cancer, according to *H. pylori* infection, smoking, tumour site and histological type.

## Materials and methods

We conducted a case–control study of incident cases of gastric adenocarcinoma admitted to the surgery wards of the two major public hospitals for cancer patients in the North of Portugal (Hospital de S. João and Instituto Português de Oncologia Francisco Gentil, both in Porto), with appropriate community controls selected among Porto dwellers. From June 2001 to December 2006, we evaluated 709 incident cases of gastric adenocarcinoma. As previously described (Lunet *et al*, 2006), patients were admitted to the surgery wards and the interview took place during in-hospital stay, shortly after admission, mostly before surgical treatment. Subjects were eligible if there was no previous cancer diagnosis and no sub-total gastrectomy for benign conditions. Cancer was diagnosed according to the routine procedures of both institutions, based on gastrectomy specimens, endoscopic biopsy material or the evaluation of metastases. Gastrectomy specimens were classified as intestinal or diffuse (Laurén, 1965). To ensure a standard pathologic classification, a single experienced pathologist reviewed all pathology reports, and slides were reassessed by three pathologists whenever routine information was considered insufficient or inconsistent, allowing reclassification according to the Laurén criteria. Anatomic location was classified following image or pathology descriptions, as cardia (defined as cardioesophageal junction, oesophagogastric junction and gastroesophageal junction) (Sobin and Wittekind, 2002) or non-cardia (all other specified sites).

Controls were part of a representative sample of the adult population of Porto. As previously described in detail (Ramos *et al*, 2004; Gal *et al*, 2005), participants were recruited by random digit dialling using households as the sampling frame, followed by simple random sampling to select one eligible person among permanent residents in each household who was invited to visit our department for interview. The overall sample comprised 2485 community controls, aged 18–92 years, corresponding to a participation proportion of 70%. Subjects were eligible for this analysis if there was no previous cancer diagnosis.

Trained interviewers inquired both cases and controls using a structured questionnaire covering demographic, social, behavioural and medical characteristics. To assess cognitive function, all individuals aged >64 years had a Mini Mental State Examination, which resulted in the exclusion of 40 cases and 20 controls scoring <18 (Murden *et al*, 1991). Dietary habits were recorded using a semiquantitative food frequency questionnaire (FFQ) comprising 82 food items/groups or beverage categories, previously validated (Lopes, 2000; Lopes *et al*, 2007), for the previous year, or the year before onset of symptoms for cases. A total of 31 cases declared to have changed their food intake ⩾12 months before interview because of gastrointestinal symptoms, and were excluded from this analysis. Cases and controls who had modified their habits during the previous year because of any other previous condition were not excluded, but were asked to recall dietary intake in the year before the change.

A blood sample was drawn and serum was kept frozen at −20 °C, as previously described (Peleteiro *et al*, 2010). Anti-*H. pylori* serum IgG titres were quantified by ELISA (Pyloriset EIA-G III (IgG); Orion Diagnostica, Espoo, Finland). Participants were classified as negative if they had <16 RU ml^–1^, as borderline if their antibody concentration was between 16 and 22 RU ml^–1^ and as positive if this was ⩾22 RU ml^–1^, according to the manufacturer's instructions. For analysis, subjects with borderline IgG titres were classified as infected. Further testing of *H. pylori* infection status was performed by western blot (Helico Blot 2.1; Genelabs Diagnostics, Singapore) in a randomly selected subsample of cases (*n*=406) and controls (*n*=255), according to the prevalence of *H. pylori* infection in each of these groups and by age strata (18–39, 40–54, 55–70 and >70 years), as determined by ELISA. The assay followed the manufacturer's instructions, and the results the recommended criteria for *H. pylori* seropositivity: (1) presence of the 116 kD band (CagA) with one or more of the following bands: 89 kD (VacA), 37, 35 or 30 kD (UreA) and 19.5 kD together, or with current infection marker; (2) presence of any band at 89, 37 or 35 kD, with or without current infection marker; and (3) presence of both 30 and 19.5 kD bands, with or without current infection marker. The presence of the CagA band was also considered to define infection, regardless of the remaining criteria. For analysis, *H. pylori* infection status was defined according to the results of the ELISA test performed in all participants, and subjects were classified as *H. pylori* negative or *H. pylori* positive. To define *H. pylori* virulence status, the results from ELISA and western blot analyses were used. A subject was classified as *H. pylori* negative if tested negative by both tests, or *H. pylori* positive if tested positive by any of the methods. CagA infection status was determined by the presence of the CagA band in the western blot evaluation, and *H. pylori-*positive subjects were divided into CagA negative and CagA positive. Information was recorded on smoking habits, and participants were classified as never smokers (those who never smoked) or ever smokers (of any amount of cigarettes).

We used different approaches to evaluate salt intake. First, we considered the sodium intake estimated using the FFQ, considering the food's intrinsic sodium content plus an estimate of added salt during cooking, taking into account the specific contribution of the different food items/groups. The database used was the Food Processor Plus software (1997; ESHA Research, Salem, OR, USA), which has been adapted to traditional Portuguese food and dishes presented in the Portuguese table of food composition (Ferreira and Graça, 1985). Second, we used information on the main food items or food groups contributing to the dietary sodium intake, according to a previous survey also conducted in the North of Portugal (Lopes *et al*, 2006), namely grains, cereals and potatoes (31.2%), particularly ‘rice, pasta and potatoes’ (14.7%) and bread (14.0%); also, ‘meat, meat products and offals’ (16.6%), ‘vegetable soup’ (15.8%), fish (12.8%) and cheese (6.7%). The consumption of these salty foods was estimated by adding up the amounts of each single item or group consumed *per* day. For analysis, the tertiles of sodium or salty foods intake observed in the controls were used as cutoffs to define groups of exposure. Dietary salt intake was also estimated through a 10-cm (3.94 inches) visual analogical scale limited by the expressions ‘without salt’ and ‘salty’, on the left and right sides of the rule, respectively. The tertiles observed in the controls were used as cutoffs to classify each subject regarding salt intake using this method.

Use of table salt was assessed by the question ‘Do you add salt to already seasoned food, at the table?’ The answer options were ‘never’, ‘sometimes’, ‘most times’ and ‘always’, and the latter three were further grouped for analysis. Subjects were asked if they had refrigerator at home, and for how many years. Duration of refrigerator ownership was categorised into <25 (including subjects who never owned a refrigerator), 25–50 and >50 years (including life-long ownership, regardless of their age).

Although all participants were asked to fill the FFQ, more specific questions regarding salt intake, namely the visual analogical scale, use of table salt and refrigerator ownership, were only performed to all cases and a subsample of controls. Data were available for 422 cases and 649 controls. No significant differences were found between subjects included and excluded from these analyses regarding gender (proportion of men: cases, 60.7 *vs* 59.1%, *P*=0.687; controls, 38.1 *vs* 38.0%, *P*=0.973). Compared with subjects with incomplete information, those included were younger (median age: cases, 62 *vs* 69 years, *P*<0.001; controls, 51 *vs* 54 years, *P*<0.001) and more educated among controls (proportion of subjects with >9 schooling years: cases, 5.7 *vs* 5.1%, *P*=0.726; controls: 45.0 *vs* 36.9%, *P*<0.001) than the excluded.

### Statistical analysis

The association between salt consumption and gastric cancer was quantified using crude and gender-, age-, education-, smoking-, *H. pylori* infection- and total energy intake-adjusted odds ratios (ORs) and the corresponding 95% confidence intervals (95% CIs), computed by unconditional logistic regression. Stratified analyses were performed according to *H. pylori* infection and virulence of the infecting strains, smoking status, tumour site and histological type. A potential effect modification by *H. pylori* or smoking was assessed by including interaction terms in the regression models, for infection status (negative or positive), virulence of the infecting strains (*H. pylori* negative, CagA negative or CagA positive) and smoking status (never or ever). In addition to the comparisons with the control group, ORs and 95% CIs were computed by unconditional logistic regression to compare gastric cancer cases according to tumour site (non-cardia *vs* cardia) and histological type (intestinal *vs* diffuse). STATA, version 9.2 (StatCorp LP, College Station, TX, USA), was used for all the analyses.

The ethics committees of involved hospitals approved the study, and all participants provided written informed consent.

## Results

Most cancers were located in the non-cardia region (86.5%) with histological type classified as intestinal (64.3%). The prevalence of *H. pylori* infection was 85.8% in cases and 85.0% in controls, as determined by ELISA, raised to 91.4% in cases and controls when western blot results are also taken into account. Among the infected, infection with CagA-positive strains was more frequent in cases (95.1%) than in controls (71.2%). The proportion of ever smokers was 43.1% among cases and 46.2% among controls.

The median sodium intake from FFQ was higher for cases than controls (3531.1 *vs* 3474.1 mg day^–1^), as well as the median number of daily servings of food items with the highest contribution to sodium intake (7.8 *vs* 7.2 servings *per* day), and the median salt consumption by visual scale (50 *vs* 45 mm). Among cases, 13.0% declared never using table salt, in comparison with 10.6% of controls. In comparison with 60.1% of controls, 34.8% of cases reported living in a house with a refrigerator for >50 years.

Risk was higher in participants with the highest salt exposures (3rd *vs* 1st third), estimated through sodium intake (OR=2.01; 95% CI: 1.16–3.46), consumption of food items with the highest contribution to sodium intake (OR=2.54; 95% CI: 1.56–4.14) and dietary salt intake evaluated by a visual scale (OR=1.83; 95% CI: 1.28–2.63). No significant association was found with frequent use of table salt (OR=1.35, 95% CI: 0.85–2.15). Subjects owning a refrigerator for a longer time had a lower gastric cancer risk (>50 *vs* <25 years, OR=0.28, 95% CI: 0.14–0.57; Table 1).

Despite the suggestion of a stronger association with salt exposure among the *H. pylori* infected (Table 2), significant differences between strata were observed only when FFQ information was used (e.g., highest *vs* lowest third of sodium intake: OR=0.67, 95% CI: 0.13–3.48 among the *H. pylori* negative and OR=2.36, 95% CI: 1.32–4.22 in the *H. pylori* positive; *P* for interaction=0.045), but no consistent differences were observed for other measures of salt exposure. When further dividing the *H. pylori* infected by virulence (Table 3), no significant differences in gastric cancer risk were observed (e.g., highest *vs* lowest third of sodium intake from FFQ: OR=4.50, 95% CI: 0.18–112.64 among the *H. pylori-*negative group, and OR=3.93, 95% CI: 0.30–52.27 in the *H. pylori-*positive/CagA-negative group, *P* for interaction=0.612; and OR=1.52, 95% CI: 0.71–3.28 in the *H. pylori-*positive/CagA-positive group, *P* for interaction=0.407).

No effect modification by smoking was observed across the different measures of dietary salt intake, the risk in never and ever smokers being similar (e.g., highest *vs* lowest third of sodium intake from FFQ: OR=1.84, 95% CI: 0.85–4.02 among never smokers and OR=2.14, 95% CI: 0.98–4.65 among ever smokers; *P* for interaction=0.960; Table 4).

No consistent or meaningful variations in gastric cancer risk associated with dietary salt intake were observed by tumour site (e.g., highest *vs* lowest third of sodium intake from FFQ: OR=1.46, 95% CI: 0.50–4.33 for non-cardia *vs* cardia cancers; Table 5) or histological type corresponding (OR=0.68, 95% CI: 0.23–2.02 for intestinal *vs* diffuse tumours; Table 6).

## Discussion

Dietary salt intake, as assessed by different methods, independently increased the risk of gastric cancer, regardless of tumour site and histological type and with no evidence of effect modification by *H. pylori* infection or smoking. Most observational studies have used questionnaires to assess dietary salt intake, with relative risk estimates ranging from 0.53 to 24.92 (Tsugane, 2005; Tsugane and Sasazuki, 2007; Wang *et al*, 2009). In our study, the odds of gastric cancer were approximately twice as high for subjects in the highest than the lowest categories of salt exposure, as estimated through sodium intake, consumption of food items with the highest contribution to sodium intake and dietary salt intake evaluated by a visual scale. Reasons for these discrepancies include: first, dietary habits in a specific setting can influence not only sodium consumption but also the perceived salt intake, and levels reported as ‘high’ in one study might be considered ‘low’ in other studies in settings with higher average exposures. Second, the public perception of deleterious effects of salt may produce a Hawthorne effect. Third, co-morbidities associated with a recommended reduction in dietary salt may not be taken into account. Fourth, recall bias may occur in case–control studies. Cases may provide a less accurate report of their past dietary habits, because unnoticed changes in intake may occur as cancer develops and becomes symptomatic (Botterweck *et al*, 1998); cancer patients may also over-report exposures perceived as causal.

A major concern in assessing salt intake are the facts that it is a natural component of most foods, and that it can also be added during cooking or at the table in amounts that people usually ignore or are unable to report accurately (Chen *et al*, 1990). Excretion of sodium in urine over a 24-h period reflects accurately the sodium ingested from different sources (World Cancer Research Fund/American Institute for Cancer Research, 2007), but it cannot be used retrospectively in case–control studies. We therefore used different approaches and in our analysis, both intrinsic sodium content and salt added during food preparation were taken into account.

Methods of measuring the salt that is added during food preparation depend not only on subjects’ perception but also on their cognitive ability for understanding and properly assigning their preference for salt use (Gagliese *et al*, 2005; Pesonen *et al*, 2009). This may explain the inconsistency in our results for the visual scale, despite the exclusion of subjects classified as cognitively impaired. Nevertheless, we found a similar positive association when assessing salt intake by the visual scale, suggesting a suitable assessment. We excluded all subjects who reported to have changed their dietary habits because of any previous condition, as co-morbidities for which a reduction in dietary salt is recommended may be a source of confounding.

The subjects analysed were younger and more educated than those excluded because of missing items (e.g., visual analogical scale, use of table salt, duration of refrigerator ownership). This may have resulted in selection bias towards the null, as, among the controls, the younger and more educated participants reported a higher salt consumption, and the positive association with salt may be underestimated in our study.

Refrigeration enables consumption of fresh foods including seasonal vegetables and fruits all year round as well as fresh meat, and reduces the need for salting, smoking, curing and pickling to preserve food. As in Portugal the widespread availability of domestic refrigeration occurred mostly at the end of the twentieth century, duration of refrigerator ownership may best reflect the length of time exposed to salt-preserved foods due more to lack of alternatives than a natural preference for these foods. Although refrigerators were initially restricted to higher social classes, leading to a potential confounding by socioeconomic status, in the present study the strong protective effect of refrigerator ownership was independent of the most probable confounders.

The increased risk in gastric cancer might be because of compounds other than salt that are produced during the preservation process. Foods such as processed meat, cured meat or dried fish, whose consumption is used as a surrogate for salt exposure, also have a high content of nitrosated compounds, which may be involved in gastric carcinogenesis (World Cancer Research Fund/American Institute for Cancer Research, 2007). In our study, using measurements less subject to the confounding effect of nitrosated compounds, such as the visual scale of salt intake, an increased risk was still observed, supporting the independent deleterious effect of salt consumption.

In our study, confounding is unlikely to be a major concern, as the main potential confounders were taken into account. Further adjustment for fruit and vegetables intake, and total or red/processed meat consumption did not change the conclusions (data not shown), and therefore we opted to show models adjusted only for gender, age, education, smoking, *H. pylori* infection and total energy intake. No information on physical activity or body mass index was collected, and although associations with these exposures show less consistent results than for the main potential confounders (Friedenreich *et al*, 2010; Gonzalez and Riboli, 2010), it is unlikely that their inclusion would yield substantially different OR estimates and different conclusions.

Proposed mechanisms by which salt can cause gastric cancer are either direct damage to the gastric mucosa leading to hyperplasia of the gastric pit epithelium with increased potential for mutations or the result of interaction with *H. pylori*, as the damage caused by salt may also increase gastric *H. pylori* colonisation (Fox *et al*, 1999; Nozaki *et al*, 2002). This would imply a stronger association among *H. pylori-*infected subjects, especially those infected with CagA-positive strains, but this was not confirmed in our study. The high prevalence of infection in Portugal compared with other Western countries (Quina, 1994) may further obscure an effect modification, as there is a high potential for misclassification of infection status, especially among the cases (Peleteiro *et al*, 2010). The possible interaction may be more important in relation to strain virulence, as infection with CagA-positive strains is a better marker of gastric cancer risk (Peleteiro *et al*, 2010). Nevertheless, it is also possible that the joint effect of salt and infection is less pronounced in our population, as infection with more virulent strains would be expected to be more important in gastric cancer risk than salt exposure. A synergistic effect might occur if any gastric mucosa damage by salt was enhanced by tobacco carcinogens (Iwata *et al*, 1995), but no effect modification was observed across the different measures of salt intake.

The World Health Organization recommendation for maximum intake of salt of 5 g day^–1^ (World Health Organization, 2003lies well below the estimated dietary intake of 9.2 g day^–1^ of the Portugese population (Lopes *et al*, 2006). One of the major sources of this dietary salt intake, taking into account only the intrinsic sodium content, is bread, which represents 24.5% of the overall sodium intake (Lopes *et al*, 2006). In August 2010, a new law was implemented in Portugal regulating the maximum quantity of salt allowed to 1.4 g per 100 g of bread (Lei no. 75/2009, 2009). This may contribute to reducing (Lunet *et al*, 2004) the risk of gastric cancer in a country with gastric cancer mortality ranking among the highest in Europe.

Taking into account site, histological type, smoking and *H. pylori* infection status and virulence, our study confirms the association between dietary salt intake and gastric cancer.

## Figures and Tables

**Table 1:** Association between gastric cancer and dietary salt intake using different approaches to evaluate salt intake

	**Controls (*n*=649)**	**Cases (*n*=422)**	**OR (95% CI)**	
	***n* (%)**	***n* (%)**	**Crude**	**Adjusted^a^**
*Sodium intake from food frequency questionnaire (mg day*^*–1*^)^b^				
<3067.5	214 (33.0)	132 (31.3)	1	1
3067.5–3960.1	215 (33.1)	146 (34.6)	1.10 (0.81–1.48)	1.39 (0.94–2.06)^c^
>3960.1	220 (33.9)	144 (34.1)	1.06 (0.78–1.44)	2.01 (1.16–3.46)^c^
				
*Food items with the highest contribution to sodium intake (servings per day)* b				
<6.3	214 (33.0)	92 (21.8)	1	1
6.3–8.1	215 (33.1)	137 (32.5)	1.48 (1.07–2.05)	1.28 (0.85–1.92)^c^
>8.1	220 (33.9)	193 (45.7)	2.04 (1.49–2.79)	2.54 (1.56–4.14)^c^
				
*Salt consumption by visual analogical scale (mm)* b				
<35	216 (33.2)	108 (25.6)	1	1
35–49	210 (32.4)	81 (19.2)	0.77 (0.55–1.09)	0.79 (0.53–1.20)
>49	223 (34.4)	233 (55.2)	2.09 (1.56–2.81)	1.83 (1.28–2.63)
				
*Use of table salt*				
Never	580 (89.4)	367 (87.0)	1	1
Sometimes/most times/always	69 (10.6)	55 (13.0)	1.26 (0.86–1.84)	1.35 (0.85–2.15)
				
*Duration of refrigerator ownership (years)*				
<25^d^	15 (2.3)	72 (17.1)	1	1
25–50	244 (37.6)	203 (48.1)	0.17 (0.10–0.31)	0.18 (0.09–0.35)
>50 (including all their lives)	390 (60.1)	147 (34.8)	0.08 (0.04–0.14)	0.28 (0.14–0.57)

Abbreviations: OR=odds ratio; CI=confidence interval.

a Adjusted for gender, age, education, smoking and *H. pylori* infection.

b Tertiles observed in controls used as cutoffs.

c Additionally adjusted for total energy intake.

d Including four cases who do not currently have refrigerator.

**Table 2:** Association between gastric cancer and the main food items/groups contributing to the dietary salt intake and the preference for salty/salted foods, according to *H. pylori* infection (ELISA)

	***H. pylori* negative (*n*=157)**				***H. pylori* positive (*n*=914)**				
	**Controls (*n*=97)**	**Cases (*n*=60)**	**OR (95% CI)**		**Controls (*n*=552)**	**Cases (*n*=362)**	**OR (95% CI)**		
	***n* (%)**	***n* (%)**	**Crude**	**Adjusted^a^**	***n* (%)**	***n* (%)**	**Crude**	**Adjusted^a^**	***P*-value^b^**
*Sodium intake from food frequency questionnaire (mg day*^*–1*^)^c^									
<3067.5	24 (24.7)	24 (40.0)	1	1	190 (34.4)	108 (29.8)	1	1	
3067.5–3960.1	33 (34.0)	21 (35.0)	0.64 (0.29–1.40)	0.93 (0.32–2.71)^d^	182 (33.0)	125 (34.5)	1.21 (0.87–1.68)	1.48 (0.96–2.26)^d^	0.470
>3960.1	40 (41.2)	15 (25.0)	0.38 (0.16–0.85)	0.67 (0.13–3.48)^d^	180 (32.6)	129 (35.7)	1.26 (0.91–1.75)	2.36 (1.32–4.22)^d^	0.045
									
*Food items with the highest contribution to sodium intake (servings per day)* c									
<6.3	25 (25.8)	16 (26.6)	1	1	189 (34.2)	76 (21.0)	1	1	
6.3–8.1	38 (39.2)	19 (31.7)	0.78 (0.33–1.80)	0.90 (0.28–2.87)^d^	177 (32.1)	118 (32.6)	1.66 (1.16–2.36)	1.35 (0.87–2.09)^d^	0.391
>8.1	34 (35.0)	25 (41.7)	1.15 (0.51–2.59)	1.67 (0.40–6.90)^d^	186 (33.7)	168 (46.4)	2.25 (1.60–3.15)	2.69 (1.60–4.54)^d^	0.178
									
*Salt consumption by visual analogical scale (mm)* c									
<35	38 (39.2)	15 (25.0)	1	1	178 (32.2)	93 (25.7)	1	1	
35–48	29 (29.9)	9 (15.0)	0.79 (0.30–2.05)	1.67 (0.46–6.04)	181 (32.8)	72 (19.9)	0.76 (0.52–1.10)	0.73 (0.47–1.12)	0.367
>48	30 (30.9)	36 (60.0)	3.04 (1.41–6.56)	4.92 (1.61–14.99)	193 (35.0)	197 (54.4)	1.95 (1.42–2.69)	1.63 (1.10–2.40)	0.142
									
*Use of table salt*									
Never	87 (86.7)	52 (86.7)	1	1	493 (89.3)	315 (87.0)	1	1	
Sometimes/most times/always	10 (10.3)	8 (13.3)	1.34 (0.50–3.61)	1.46 (0.39–5.48)	59 (10.7)	47 (13.0)	1.25 (0.83–1.88)	1.31 (0.80–2.16)	0.862
									
*Duration of refrigerator ownership (years)*									
<25^e^	1 (1.0)	15 (25.0)	1	1	14 (2.6)	57 (15.8)	1	1	
25–50	18 (18.6)	22 (36.7)	0.08 (0.01–0.68)	0.13 (0.01–1.14)	226 (40.9)	181 (50.0)	0.20 (0.11–0.36)	0.18 (0.09–0.38)	0.807
>50 (including all their lives)	78 (80.4)	23 (38.3)	0.02 (0.00–0.16)	0.14 (0.02–1.28)	312 (56.5)	124 (34.2)	0.10 (0.05–0.18)	0.31 (0.15–0.65)	0.613

Abbreviations: ELISA=enzyme-linked immunosorbent assay; OR=odds ratio; CI=confidence interval.

a Adjusted for gender, age, education and smoking.

b *P*-value for interaction.

c Tertiles observed in controls used as cutoffs.

d Additionally adjusted for total energy intake.

e Including four cases who do not currently have refrigerator.

**Table 3:** Association between gastric cancer and the main food items/groups contributing to the dietary salt intake and the preference for salty/salted foods, according to *H. pylori* virulence (ELISA and western blot)

	***H. pylori* negative (*n*=57)**				***H. pylori* positive/CagA negative (*n*=85)**					***H. pylori* positive/CagA positive (*n*=519)**				
	**Controls (*n*=22)**	**Cases (*n*=35)**	**OR (95% CI)**		**Controls (*n*=67)**	**Cases (*n*=18)**	**OR (95% CI)**			**Controls (*n*=166)**	**Cases (*n*=353)**	**OR (95% CI)**		
	***n* (%)**	***n* (%)**	**Crude**	**Adjusted^a^**	***n* (%)**	***n* (%)**	**Crude**	**Adjusted^a^**	***P*-value^b^**	***n* (%)**	***n* (%)**	**Crude**	**Adjusted^a^**	***P*-value^b^**
*Sodium intake from food frequency questionnaire (mg day*^*–1*^)^c^														
<3067.5	5 (22.7)	12 (34.2)	1	1	28 (41.8)	7 (38.9)	1	1		58 (35.0)	104 (29.5)	1	1	
3067.5–3960.1	8 (36.4)	15 (42.9)	0.78 (0.20–3.02)	2.56 (0.33–19.84)^d^	21 (31.3)	8 (44.4)	1.52 (0.48–4.87)	2.15 (0.40–11.48)^d^	0.803	54 (32.5)	119 (33.7)	1.23 (0.78–1.94)	1.31 (0.75–2.30)^d^	0.647
>3960.1	9 (40.9)	8 (22.9)	0.37 (0.09–1.52)	4.50 (0.18–112.64)^d^	18 (26.9)	3 (16.7)	0.67 (1.15–2.92)	3.93 (0.30–52.27)^d^	0.612	54 (32.5)	130 (36.8)	1.34 (0.85–2.11)	1.52 (0.71–3.28)^d^	0.407
														
*Food items with the highest contribution to sodium intake (servings per day)* c														
<6.3	2 (9.1)	9 (25.7)	1	1	24 (35.8)	7 (38.9)	1	1		60 (36.2)	70 (19.8)	1	1	
6.3–8.1	12 (54.5)	10 (28.6)	0.18 (0.03–1.06)	0.15 (0.01–1.92)^d^	23 (34.3)	3 (16.7)	0.45 (0.10–1.94)	0.84 (0.11–6.23)^d^	0.569	48 (28.9)	119 (33.7)	2.12 (1.31–3.44)	1.56 (0.88–2.74)^d^	0.078
>8.1	8 (36.4)	16 (45.7)	0.44 (0.08–2.56)	0.30 (0.02–5.09)^d^	20 (29.9)	8 (44.4)	1.37 (0.42–4.44)	14.46 (1.30–161.22)^d^	0.081	58 (34.9)	164 (46.5)	2.42 (1.54–3.83)	2.24 (1.14–4.42)^d^	0.060
														
*Salt consumption by visual analogical scale (mm)* c														
<35	9 (40.9)	9 (25.8)	1	1	20 (29.9)	6 (33.3)	1	1		49 (29.5)	88 (24.9)	1	1	
35–48	6 (27.3)	6 (17.1)	1.00 (0.23–4.31)	1.27 (0.14–11.53)	24 (35.8)	5 (27.8)	0.69 (0.18–2.62)	0.32 (0.06–1.82)	0.456	60 (36.1)	69 (19.6)	0.64 (0.39–1.05)	0.67 (0.38–1.17)	0.732
>48	7 (31.8)	20 (57.1)	2.86 (0.81–10.10)	9.68 (1.14–81.83)	23 (34.3)	7 (38.9)	1.01 (0.29–3.52)	0.36 (0.07–1.94)	0.084	57 (34.4)	196 (55.5)	1.91 (1.21–3.02)	1.69 (1.00–2.85)	0.453
														
*Use of table salt*														
Never	19 (86.4)	30 (85.7)	1	1	63 (94.0)	17 (94.4)	1	1		154 (92.8)	305 (86.4)	1	1	
Sometimes/most times/always	3 (13.6)	5 (14.3)	1.06 (0.22–4.94)	2.16 (0.18–26.67)	4 (6.0)	1 (5.6)	0.93 (0.10–8.84)	3.44 (0.06–181.33)	0.562	12 (7.2)	48 (13.6)	2.02 (1.04–3.91)	1.81 (0.86–3.78)	0.485
														
*Duration of refrigerator ownership (years)*														
<25^e^	1 (4.5)	9 (25.7)	1	1	0 (0.0)	4 (22.2)	1	1		7 (4.2)	57 (16.2)	1	1	
25–50	4 (18.2)	11 (31.4)	0.30 (0.03–3.24)	0.29 (0.02–4.37)	28 (41.8)	10 (55.6)	—	—	—	81 (48.8)	177 (50.1)	0.27 (0.12–0.61)	0.29 (0.12–0.70)	0.796
>50 (including all their lives)	17 (77.3)	15 (42.9)	0.10 (0.01–0.87)	1.67 (0.10–27.36)	39 (58.2)	4 (22.2)	—	—	—	78 (47.0)	119 (33.7)	0.19 (0.08–0.43)	0.47 (0.19–1.17)	0.918

Abbreviations: ELISA=enzyme-linked immunosorbent assay; OR=odds ratio; CI=confidence interval.

a Adjusted for gender, age, education and smoking.

b *P*-value for interaction.

c Tertiles observed in controls used as cutoffs.

d Additionally adjusted for total energy intake.

e Including two cases who do not currently have refrigerator.

**Table 4:** Association between gastric cancer and the main food items/groups contributing to the dietary salt intake and the preference for salty/salted foods, according to smoking status

	**Never smokers (*n*=589)**				**Ever smokers (*n*=482)**				
	**Controls (*n*=349)**	**Cases (*n*=240)**	**OR (95% CI)**		**Controls (*n*=300)**	**Cases (*n*=182)**	**OR (95% CI)**		
	***n* (%)**	***n* (%)**	**Crude**	**Adjusted^a^**	***n* (%)**	***n* (%)**	**Crude**	**Adjusted^a^**	***P*-value^b^**
*Sodium intake from food frequency questionnaire (mg day*^*–1*^)^c^									
<3067.5	128 (36.7)	89 (37.1)	1	1	86 (28.7)	43 (23.6)	1		
3067.5–3960.1	117 (33.5)	89 (37.1)	1.09 (0.74–1.61)	1.40 (0.84–2.34)^d^	98 (32.6)	57 (31.3)	1.16 (0.71–1.90)	1.31 (0.69–2.48)^d^	0.734
>3960.1	104 (29.8)	62 (25.8)	0.86 (0.57–1.30)	1.84 (0.85–4.02)^d^	116 (38.7)	82 (45.1)	1.41 (0.89–2.24)	2.14 (0.98–4.65)^d^	0.960
									
*Food items with the highest contribution to sodium intake (servings per day)* c									
<6.3	132 (37.8)	65 (27.1)	1	1	82 (27.3)	27 (14.8)	1	1	
6.3–8.1	108 (31.0)	81 (33.7)	1.52 (1.01–2.30)	1.52 (0.91–2.54)^d^	107 (35.7)	56 (30.8)	1.59 (0.92–2.73)	0.91 (0.45–1.82)^d^	0.198
>8.1	109 (31.2)	94 (39.2)	1.75 (1.17–2.63)	2.64 (1.38–5.03)^d^	111 (37.0)	99 (54.4)	2.71 (1.62–4.52)	2.19 (1.01–4.73)^d^	0.542
									
*Salt consumption by visual analogical scale (mm)* c									
<35	121 (34.7)	71 (29.6)	1	1	95 (31.7)	37 (20.3)	1	1	
35–48	120 (34.4)	48 (20.0)	0.68 (0.44–1.06)	0.71 (0.42–1.20)	90 (30.0)	33 (18.1)	0.94 (0.54–1.63)	0.94 (0.48–1.84)	0.648
>48	108 (30.9)	121 (50.4)	1.91 (1.29–2.82)	1.75 (1.09–2.80)	115 (38.3)	112 (61.6)	2.50 (1.58–3.96)	2.00 (1.12–3.56)	0.933
									
*Use of table salt*									
Never	314 (90.0)	216 (90.0)	1	1	266 (88.7)	151 (83.0)	1	1	
Sometimes/most times/always	35 (10.0)	24 (10.0)	1.00 (0.58–1.72)	1.04 (0.52–2.04)	34 (11.3)	31 (17.0)	1.61 (0.95–2.72)	1.64 (0.86–3.12)	0.406
									
*Duration of refrigerator ownership (years)*									
<25^e^	9 (2.6)	53 (22.1)	1	1	6 (2.0)	19 (10.4)	1	1	
25–50	157 (45.0)	121 (50.4)	0.13 (0.06–0.28)	0.14 (0.06–0.32)	87 (29.0)	82 (45.1)	0.30 (0.11–0.78)	0.36 (0.11–1.14)	0.134
>50 (including all their lives)	183 (52.4)	66 (27.5)	0.06 (0.03–0.13)	0.20 (0.08–0.46)	207 (69.0)	81 (44.5)	0.12 (0.05–0.32)	0.69 (0.22–2.21)	0.090

Abbreviations: OR=odds ratio; CI=confidence interval.

a Adjusted for gender, age, education and *H. pylori* infection.

b *P*-value for interaction.

c Tertiles observed in controls used as cutoffs.

d Additionally adjusted for total energy intake.

e Including four cases who do not currently have refrigerator.

**Table 5:** Association between gastric cancer and the main food items/groups contributing to the dietary salt intake and the preference for salty/salted foods according to tumour site

		**Cases (*n*=379)**							
	**Controls (*n*=649)**	**Non-cardia (*n*=328)**	**OR (95% CI)**		**Cardia (*n*=51)**	**OR (95% CI)**		**Non-cardia *vs* cardia OR (95% CI)**	
	***n* (%)**	***n* (%)**	**Crude**	**Adjusted^a^**	***n* (%)**	**Crude**	**Adjusted^a^**	**Crude**	**Adjusted^a^**
*Sodium intake from food frequency questionnaire (mg day*^*–1*^)^b^									
<3067.5	214 (33.0)	100 (30.5)	1	1	14 (27.5)	1	1	1	1
3067.5–3960.1	215 (33.1)	115 (35.1)	1.14 (0.82–1.59)	1.51 (0.99–2.30)^c^	20 (39.2)	1.42 (0.70–2.89)	1.51 (0.68–3.35)^c^	0.80 (0.39–1.68)	0.94 (0.43–2.06)^c^
>3960.1	220 (33.9)	113 (34.4)	1.10 (0.79–1.53)	2.26 (1.27–4.04)^c^	17 (33.3)	1.18 (0.56–2.46)	1.50 (0.50–4.52)^c^	0.93 (0.44–1.98)	1.46 (0.50–4.33)^c^
									
*Food items with the highest contribution to sodium intake (servings per day)* b									
<6.3	214 (33.0)	69 (21.0)	1	1	12 (23.5)	1	1	1	1
6.3–8.1	215 (33.1)	104 (31.7)	1.50 (1.05–2.15)	1.32 (0.85–2.04)^c^	18 (35.3)	1.49 (0.70–3.18)	1.05 (0.46–2.37)^c^	1.00 (0.46–2.22)	1.18 (0.52–2.66)^c^
>8.1	220 (33.9)	155 (47.3)	2.18 (1.55–3.07)	3.00 (1.79–5.05)^c^	21 (41.2)	1.70 (0.82–3.54)	1.34 (0.50–3.57)^c^	1.28 (0.60–2.76)	2.08 (0.80–5.44)^c^
									
*Salt consumption by visual analogical scale (mm)* b									
<35	216 (33.2)	86 (26.2)	1	1	10 (19.6)	1	1	1	1
35–48	210 (32.4)	61 (18.6)	0.73 (0.50–1.06)	0.75 (0.48–1.16)	11 (21.6)	1.13 (0.47–2.72)	1.17 (0.47–2.93)	0.64 (0.26–1.61)	0.63 (0.25–1.61)
>48	223 (34.4)	181 (55.2)	2.04 (1.48–2.80)	1.81 (1.24–2.66)	30 (58.8)	2.90 (1.39–6.09)	2.63 (1.20–5.75)	0.70 (0.33–1.50)	0.67 (0.31–1.48)
									
*Use of table salt*									
Never	580 (89.4)	283 (86.3)	1	1	48 (94.1)	1	1	1	1
Sometimes/most times/always	69 (10.6)	45 (13.7)	1.34 (0.89–2.00)	1.46 (0.90–2.37)	3 (5.9)	0.52 (0.16–1.73)	0.55 (0.16–1.90)	2.54 (0.76–8.52)	2.66 (0.78–9.01)
									
*Duration of refrigerator ownership (years)*									
<25^d^	15 (2.3)	58 (17.7)	1	1	8 (15.7)	1	1	1	1
25–50	244 (37.6)	155 (47.3)	0.16 (0.09–0.30)	0.16 (0.08–0.33)	27 (52.9)	0.21 (0.08–0.53)	0.19 (0.07–0.53)	0.79 (0.34–1.84)	0.88 (0.37–2.11)
>50 (including all their lives)	390 (60.1)	115 (35.1)	0.08 (0.04–0.14)	0.27 (0.13–0.54)	16 (31.4)	0.08 (0.03–0.21)	0.28 (0.09–0.84)	0.99 (0.40–2.45)	0.97 (0.35–2.69)

Abbreviations: OR=odds ratio; CI=confidence interval.

a Adjusted for gender, age, education, smoking and *H. pylori* infection.

b Tertiles observed in controls used as cutoffs.

c Additionally adjusted for total energy intake.

d Including four cases who do not currently have refrigerator.

**Table 6:** Association between gastric cancer and the main food items/groups contributing to the dietary salt intake and the preference for salty/salted foods according to histological type

		**Cases (*n*=325)**							
	**Controls (*n*=649)**	**Intestinal (*n*=209)**	**OR (95% CI)**		**Diffuse (*n*=116)**	**OR (95% CI)**		**Intestinal *vs* diffuse OR (95% CI)**	
	***n* (%)**	***n* (%)**	**Crude**	**Adjusted^a^**	***n* (%)**	**Crude**	**Adjusted^a^**	**Crude**	**Adjusted^a^**
*Sodium intake from food frequency questionnaire (mg day*^*–1*^)^b^									
<3067.5	214 (33.0)	67 (32.1)	1	1	37 (31.9)	1	1	1	1
3067.5–3960.1	215 (33.1)	68 (32.5)	1.01 (0.69–1.49)	1.30 (0.79–2.14)^c^	40 (34.5)	1.08 (0.66–1.75)	1.27 (0.72–2.25)^c^	1.24 (0.60–2.59)	1.06 (0.49–2.33)^c^
>3960.1	220 (33.9)	74 (35.4)	1.07 (0.73–1.57)	2.19 (1.11–4.35)^c^	39 (33.6)	1.02 (0.63–1.67)	1.54 (0.70–3.41)^c^	1.07 (0.50–2.29)	0.68 (0.23–2.02)^c^
									
*Food items with the highest contribution to sodium intake (servings per day)* b									
<6.3	214 (33.0)	44 (21.0)	1	1	25 (21.5)	1	1	1	1
6.3–8.1	215 (33.1)	70 (33.5)	1.58 (1.04–2.41)	1.36 (0.82–2.28)^c^	40 (34.5)	1.59 (0.93–2.72)	1.36 (0.75–2.47)^c^	1.00 (0.45–2.20)	0.85 (0.38–1.92)^c^
>8.1	220 (33.9)	95 (45.5)	2.10 (1.40–3.14)	2.68 (1.45–4.97)^c^	51 (44.0)	1.98 (1.19–3.32)	2.12 (1.04–4.33)^c^	0.78 (0.36–1.67)	0.48 (0.18–1.26)^c^
									
*Salt consumption by visual analogical scale (mm)* b									
<35	216 (33.2)	58 (27.7)	1	1	30 (25.9)	1	1	1	1
35–48	210 (32.4)	39 (18.7)	0.69 (0.44–1.08)	0.73 (0.43–1.22)	22 (19.0)	0.75 (0.42–1.35)	0.70 (0.38–1.30)	1.55 (0.62–3.88)	1.58 (0.62–4.03)
>48	223 (34.4)	112 (53.6)	1.87 (1.29–2.70)	1.70 (1.09–2.65)	64 (55.1)	2.07 (1.29–3.31)	1.69 (1.01–2.84)	1.42 (0.67–3.05)	1.48 (0.68–3.26)
									
*Use of table salt*									
Never	580 (89.4)	190 (90.9)	1	1	98 (84.5)	1	1	1	1
Sometimes/most times/always	69 (10.6)	19 (9.1)	0.84 (0.49–1.43)	0.90 (0.48–1.68)	18 (15.5)	1.54 (0.88–2.71)	1.53 (0.82–2.85)	0.39 (0.12–1.32)	0.38 (0.11–1.28)
									
*Duration of refrigerator ownership (years)*									
<25^d^	15 (2.3)	33 (15.8)	1	1	26 (22.4)	1	1	1	1
25–50	244 (37.6)	120 (57.4)	0.22 (0.12–0.43)	0.22 (0.10–0.48)	38 (32.8)	0.09 (0.04–0.18)	0.10 (0.04–0.21)	1.26 (0.54–2.94)	1.13 (0.47–2.70)
>50 (including all their lives)	390 (60.1)	56 (26.8)	0.06 (0.03–0.13)	0.26 (0.12–0.58)	52 (44.8)	0.08 (0.04–0.15)	0.22 (0.10–0.48)	1.01 (0.41–2.49)	1.03 (0.37–2.86)

Abbreviations: OR=odds ratio; CI=confidence interval.

a Adjusted for gender, age, education, smoking and *H. pylori* infection.

b Tertiles observed in controls used as cutoffs.

c Additionally adjusted for total energy intake.

d Including four cases who do not currently have refrigerator.
